# Antiangiogenic agents targeting different angiogenic pathways have opposite effects on tumor hypoxia in R-18 human melanoma xenografts

**DOI:** 10.1186/s12885-017-3404-4

**Published:** 2017-06-12

**Authors:** Jon-Vidar Gaustad, Trude G. Simonsen, Lise Mari K. Andersen, Einar K. Rofstad

**Affiliations:** Group of Radiation Biology and Tumor Physiology, Department of Radiation Biology, Institute for Cancer Research, Oslo University Hospital, Oslo, Norway

**Keywords:** Malignant melanoma, Sunitinib, Properdistatin, Vascular morphology, Vascular function, Tumor hypoxia

## Abstract

**Background:**

Studies comparing the effect of antiangiogenic agents targeting different angiogenic pathways are sparse. The purpose of this study was to compare the effect of properdistatin and sunitinib treatment in a preclinical model of malignant melanoma. Properdistatin is a small peptide derived from the thrombospondin-1 domain of the plasma protein properdin, and sunitinib is a tyrosine kinase inhibitor targeting several receptors including the vascular endothelial growth factor receptors.

**Methods:**

R-18 human melanoma xenografts growing in dorsal window chambers were treated with properdistatin, sunitinib, or vehicle. Parameters describing the morphology of tumor vasculature were assessed from high-resolution transillumination images, and BST (blood supply time; the time needed for arterial blood to flow from the main supplying artery to downstream microvessels) was assessed from first-pass imaging movies recorded after a bolus of fluorescence-labeled dextran had been administered intravenously. Tumor hypoxia was assessed from immunohistochemical preparations of the imaged tissue by using pimonidazole as a hypoxia marker.

**Results:**

Properdistatin treatment selectively removed small-diameter vessels and reduced BST, whereas sunitinib treatment reduced the density of small- and large-diameter vessel similarly and did not change BST. These observations imply that properdistatin treatment reduced geometric resistance to blood flow and improved vascular function, whereas sunitinib treatment did not affect vascular function. Accordingly, sunitinib-treated tumors showed higher hypoxic fractions than properdistatin-treated tumors.

**Conclusions:**

Properdistatin and sunitinib both inhibited angiogenesis, but had distinctly different effects on vascular morphology, vascular function, and extent of hypoxia in R-18 human melanoma xenografts.

**Electronic supplementary material:**

The online version of this article (doi:10.1186/s12885-017-3404-4) contains supplementary material, which is available to authorized users.

## Background

Most tumor cells produce and secrete proteins that can stimulate or inhibit angiogenesis, and the rate of angiogenesis is given by the ratio between the angiogenic stimulators and inhibitors [1]. Different strategies to inhibit angiogenesis have been developed. These include the use of endogenous angiogenic inhibitors or small peptides that mimic these inhibitors [2], and inhibition of angiogenic stimulators or their receptors by using monoclonal antibodies or tyrosine kinase inhibitors [3–5]. There is substantial evidence that melanoma progression requires angiogenesis [6, 7]. Accordingly, several antiangiogenic agents have been tested in clinical trials but none of the agents have improved survival for patients with malignant melanoma when used as a single treatment [7]. Currently, clinical trials are evaluating whether antiangiogenic treatment in combination with immunotherapy or conventional chemotherapy can improve survival for patients with malignant melanoma [7].

The tumor microenvironment can significantly affect the outcome of conventional therapy. Poor blood supply may impair the uptake of therapeutic drugs, and tumors with extensive hypoxia are resistant to ionizing radiation, immunotherapy, and some forms of chemotherapy [8, 9]. Antiangiogenic treatments targeting the vascular endothelial growth factor A (VEGF-A) pathway have been shown to improve blood supply and oxygenation in some preclinical tumor models, and to reduce blood supply and induce hypoxia in others [3, 4, 10–12]. The reasons for these different effects are not well understood but may have substantial impact on combination therapies where antiangiogenic treatment is applied prior to or concurrent with conventional therapy [13]. It is also possible that antiangiogenic agents targeting different angiogenic pathways can affect the same tumor model differently. However studies comparing microenvironmental effects of antiangiogenic agents targeting different angiogenic pathways have not been reported thus far.

Properdistatin is a small peptide derived from the thrombospondin-1 domain of the plasma protein properdin, and is designed to mimic the endogeneous angiogenic inhibitor thrombospondin-1. Thrombospondin-1 inhibits angiogenesis by inducing apoptosis in endothelial cells by binding to the transmembrane receptor CD36 [14–16]. We have previously shown that properdistatin treatment inhibits angiogenesis in melanoma xenografts with low thrombospondin-1 expression [17]. In the current study, we investigate the effect of properdistatin on tumor oxygenation and we compare the effect of properdistatin with the effect of sunitinib treatment. Sunitinib is a tyrosine kinase inhibitor that targets several receptors including VEGF receptors 1–3, and thus inhibits angiogenesis by targeting the VEGF pathway [5]. In the current study, we show that properdistatin and sunitinib treatment affects vascular morphology and function fundamentally different, and that properdistatin-treated tumors show lower hypoxic fractions than sunitinib-treated tumors despite a similar reduction in overall vessel density.

## Methods

### Mice

Adult (8–10 weeks of age) female BALB/c *nu/nu* mice were used as host animals for dorsal window chamber preparations. The mice were bred at our institute and maintained under specific pathogen-free conditions at constant temperature (24–26 °C) and humidity (30–50%). After implantation of dorsal window chambers, the mice were kept at a temperature of 32 °C and a humidity of 60–70%. The animal experiments were approved by the Norwegian National Animal Research Authority and were done according to the Interdisciplinary Principles and Guidelines for the Use of Animals in Research, Marketing, and Education (New York Academy of Sciences, New York, NY).

### Anesthesia

Window chamber implantation and intravital microscopy examinations were carried out with anesthetized mice. Fentanyl citrate (Janssen Pharmaceutica, Beerse, Belgium), fluanisone (Janssen Pharmaceutica), and midazolam (Hoffmann-La Roche, Basel, Switzerland) were administered intraperitoneally in doses of 0.63 mg/kg, 20 mg/kg, and 10 mg/kg, respectively. After surgery, the mice were given a single injection of buprenorphine (Temgesic; Schering-Plough, Brussels, Belgium) intraperitoneally in a dose of 0.12 mg/kg to relieve pain.

### Window chamber preparations

Window chambers were implanted into the dorsal skin fold as described previously [18]. Briefly, the chamber consisted of two parallel frames, and after implantation, the frames sandwiched an extended double layer of skin. Before the chamber was implanted, a circular hole with a diameter of approximately 6.0 mm was made in one of the skin layers. A plastic window with a diameter of 6.0 mm was attached to the frame on the surgical side with a clip to provide visual access to the fascial side of the opposite skin layer. Tumors were initiated by implanting tumor specimens with a diameter of 200 to 400 μm onto the exposed skin layer. Tumor specimens were prepared from 200 to 400 mm^3^ intradermal R-18 flank tumors initiated by inoculating R-18 human melanoma cells transfected with green fluorescent protein (GFP) as described previously [19].

### Treatment

Properdistatin (Abgent, San Diego, CA) was dissolved in Hanks’ balanced salt solution (HBSS). Sunitinb L-malate (LC Laboratories, Woburn, MA) was dissolved in hydrochloric acid (1.0 M ratio of sunitinib), polysorbate 80 (0.5%; Sigma-Aldrich, Schnelldorf, Germany), polyethylene Glycol 300 (10%; Sigma-Aldrich), sodium hydroxide (to adjust pH to 3.5) and sterile water. Mice were treated with 80 mg/kg/day properdistatin or properdistatin-vehicle by intraperitoneal injections, or 40 mg/kg/day sunitinib or sunitinib-vehicle by oral administration. Tumors in mice given properdistatin-vehicle did not differ from tumors in mice given sunitinib-vehicle, and thus these tumors were pooled together and referred to as untreated tumors (vehicle). The treatments started after the tumors were vascularized and lasted for 4 days. Adverse effects were not observed with any of the treatments.

### Intravital microscopy

The mice were kept in a specially constructed holder that fixed the window chamber to the microscope stage during intravital microscopy. The body core temperature was kept at 37 to 38 °C by using a hot-air generator. Imaging was performed by using an inverted fluorescence microscope equipped with filters for green and red light (IX-71; Olympus, Munich, Germany), a black and white CCD camera (C9300–024; Hamamatsu Photonics, Hamamatsu, Japan), and appropriate image acquisition software (Wasabi; Hamamatsu Photonics). Tumor vasculature was visualized by using transillumination and filters for green light, and tumor vascular networks were mapped by recording 1–4 single frames with a × 4 objective lens resulting in a field of view of 3.80 × 3.80 mm^2^ and a pixel size of 3.7 × 3.7 μm^2^. To study the function of tumor vasculature, first-pass imaging movies were recorded after a 0.2 mL bolus of 50 mg/mL tetramethylrhodamine isothiocyanate-labeled dextran (Sigma-Aldrich) with a molecular weight of 155 kDa was injected into the lateral tail vein. First-pass imaging movies were recorded at a frame rate of 22.3 frames per second by using a × 2 objective lens, resulting in a temporal resolution of 44.8 ms, a field of view of 5.97 × 5.97 mm^2^, and a pixel size of 7.5 × 7.5 μm^2^. All recordings were stored and analyzed offline.

### Analysis of vascular morphology

Two-dimensional projected vascular masks were produced manually from transillumination images recorded with a × 4 objective lens. Vessel density (i.e., total vessel length per mm^2^ tumor area) and vessel diameter were computed from the vascular masks by algorithms implemented in MATLAB software (The MathWorks, Natick, MA), as previously described [18].

### Analysis of vascular function

Two-dimensional projected vascular masks were produced from the movies recorded with a × 2 objective lens as described previously [18]. Blood supply time (BST) images were produced by assigning a BST value to each pixel of the vascular masks. The BST of a pixel was defined as the time difference between the frame showing maximum fluorescence intensity in the pixel and the frame showing maximum fluorescence intensity in the main tumor supplying artery, as described in detail previously [20].

### Tumor hypoxia

The oxygenation status of the tumors was assessed immediately after the intravital microscopy examinations. Pimonidazole hydrochloride, dissolved in 0.9% sodium chloride, was administered intraperitoneally at a dose of 30 mg/kg. The tumors were resected approximately 4 h after pimonidazole administration and fixed in phosphate-buffered 4% paraformaldehyde. A peroxidase-based immunohistochemical assay was used to detect tumor hypoxia [21]. Histologic sections, prepared by use of standard procedures, were incubated with polyclonal rabbit antiserum to pimonidazole-protein adducts. Diaminobenzidine was used as chromogen, and hematoxylin was used for counterstaining. Hypoxic area fractions were determined by image analysis.

### Statistical analysis

Statistical comparisons of data were carried out by the Student’s t test when the data complied with the conditions of normality and equal variance. Under other conditions, comparisons were done by nonparametric analysis using the Mann-Whitney rank sum test. Probability values of *P* < 0.05, determined from two-sided tests, were considered significant. The statistical analysis was performed by using the SigmaStat statistical software (SPSS Science, Chicago, IL).

## Results

**Fig. 1:**
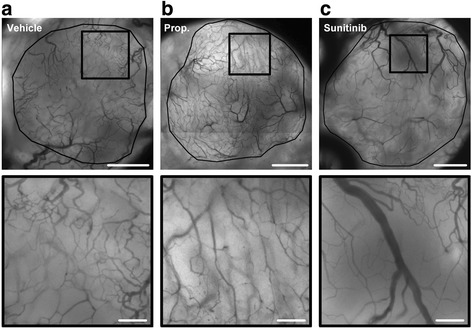
Images of tumor vasculature. Intravital microscopy images of untreated (**a**), properdistatin-treated (**b**), and sunitinib-treated R-18 tumors (**c**). Upper panels show the entire tumor vasculature and lower panels show high magnification images of the highlighted regions. Tumor area is delineated by a solid *black* line. Scale bars, 1 mm (upper panels) and 200 μm (lower panels)

**Fig. 2:**
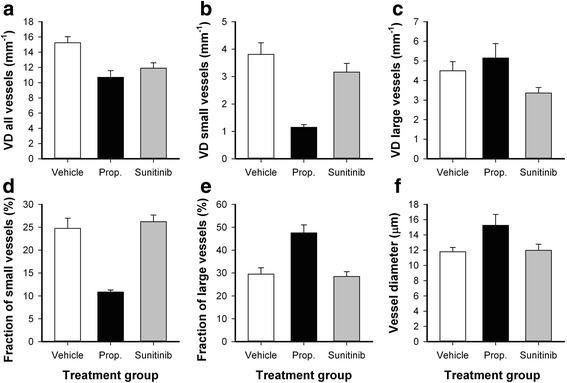
Vascular morphology. Vessel density of all vessels (**a**), vessel density of small-diameter vessels (**b**), vessel density of large-diameter vessels (**c**), fraction of small-diameter vessels (**d**), fraction of large-diameter vessels (**e**), and mean vessel diameter (**f**) in untreated, properdistain-treated, and sunitinib-treated R-18 tumors. Small-diameter vessels refer to vessels with diameter < 5 μm and large-diameter vessels refer to vessels with diameter > 15 μm. Columns, means of 5–8 tumors; bars, SE

Mice were divided in groups with matched tumor size to receive properdistatin treatment, sunitinib treatment, or no treatment (vehicle) after the tumors had developed vascular networks. Figure 1 shows intravital microscopy images of representative untreated, properdistatin-treated, and sunitinib-treated tumors. Tumors treated with properdistatin or sunitinib showed lower overall vessel density than untreated tumors (Fig. 2a; vehicle vs properdistatin: *P* = 0.004; vehicle vs sunitinib: *P* = 0.005), and properdistatin-treated tumors did not differ from sunitinib-treated tumors in overall vessel density (Fig. 2a; *P* > 0.05). In sunitinib-treated tumors, the density of small-diameter vessels (< 5 μm) and the density large-diameter vessels (>15 μm) was similarly reduced (Fig. 2b-c), and consequently, sunitinib-treated tumors did not differ from untreated tumors in the fraction of small-diameter vessels or the fraction of large-diameter vessels (Fig. 2d-e; *P* > 0.05). In properdistatin-treated tumors, the density of small-diameter vessels was significantly lower than in untreated tumors (Fig. 2b; *P* = 0.001), whereas the density of large-diameter vessels was similar in these groups (Fig. 2c; *P* > 0.05). Consequently, properdistain-treated tumors showed a lower fraction of small-diameter vessels than untreated and sunitinib-treated tumors (Fig. 2d; vehicle vs properdistatin: *P* = 0.007; sunitinib vs properdistatin: *P* = 0.004), and a higher fraction of large-diameter vessels than untreated and sunitinib-treated tumors (Fig. 2e; vehicle vs properdistatin: *P* = 0.002; sunitinib vs properdistatin: *P* < 0.001). Taken together these findings imply that properdistatin treatment selectively removed small-diameter vessels, whereas sunitinib treatment affected small- and large-diameter vessels similarily. In accordance with this implication, properdistatin-treated tumors showed larger mean vessel diameter than untreated and sunitinib-treated tumors (Fig. 2f; vehicle vs properdistatin: *P* = 0.012; sunitinib vs properdistatin: *P* = 0.037), whereas sunitinib-treated tumors did not differ from untreated tumors in mean vessel diameter (Fig. 2f; *P* > 0.05).

**Fig. 3:**
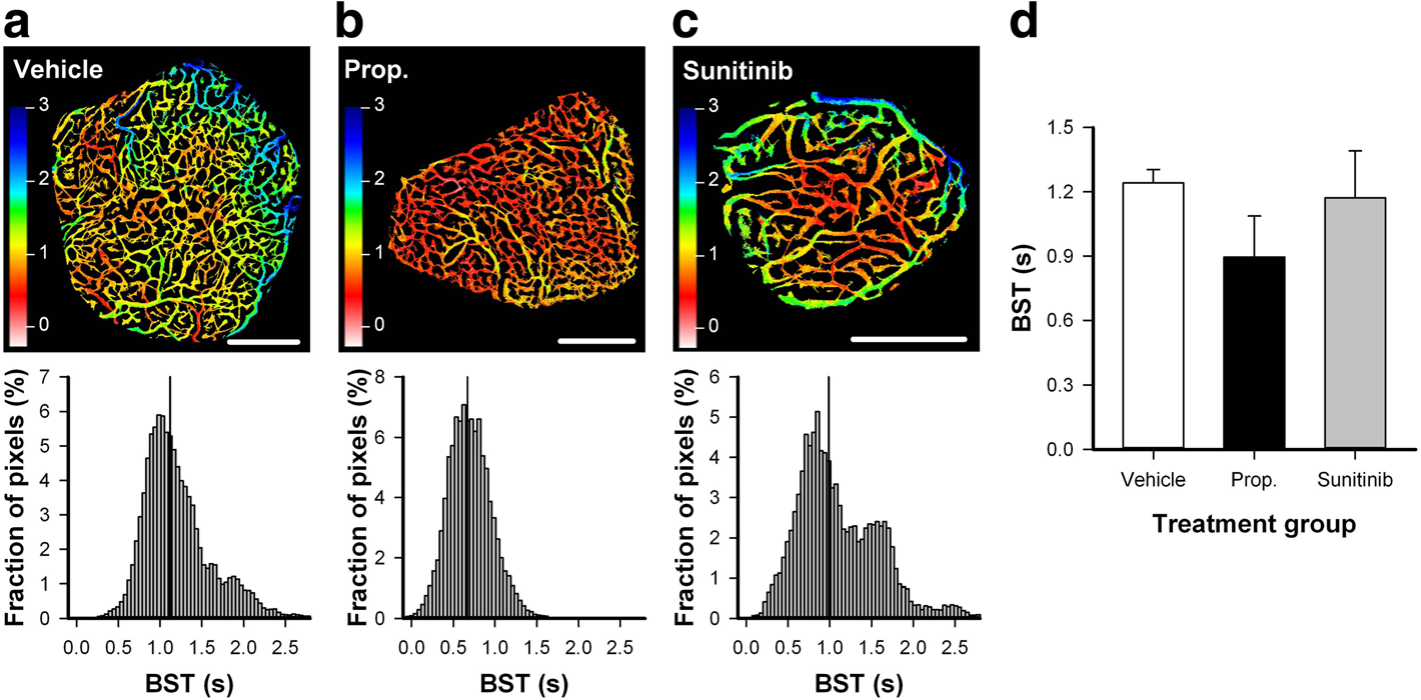
Vascular function. **a-c,** Blood supply time (BST) images and the corresponding BST frequency distributions of untreated (**a**), properdistatin-treated (**b**), and sunitinib-treated R-18 tumors (**c**). Color bars, BST scale in seconds; vertical lines, median BST; scale bars, 1 mm. The BST image of the untreated R-18 tumor (**a**) was produced from the first-pass imaging movie shown in Additional file 1. **d,** Median BST in untreated, properdistatin-treated, and sunitinib-treated R-18 tumors. Columns, means of 4–9 tumors; bars, SE

To investigate whether the treatment-induced effects on vascular morphology affected vascular function, first pass imaging movies were recorded after a bolus of fluorescence-labeled dextran had been injected into the circulation. A representative first-pass imaging movie of an untreated tumor is presented in Additional file 1. BST images and BST frequency distributions were produced from the first-pass imaging movies. Figure 3 a-c shows the BST images and the corresponding BST frequency distributions of representative untreated, properdistatin-treated, and sunitinib-treated tumors. Properdistatin-treated tumors showed significantly lower BST than untreated tumors (Fig. 3d; *P* = 0.040), whereas sunitinib-treated tumors did not differ from untreated tumors in BST (Fig. 3d; *P* > 0.05).

**Fig. 4:**
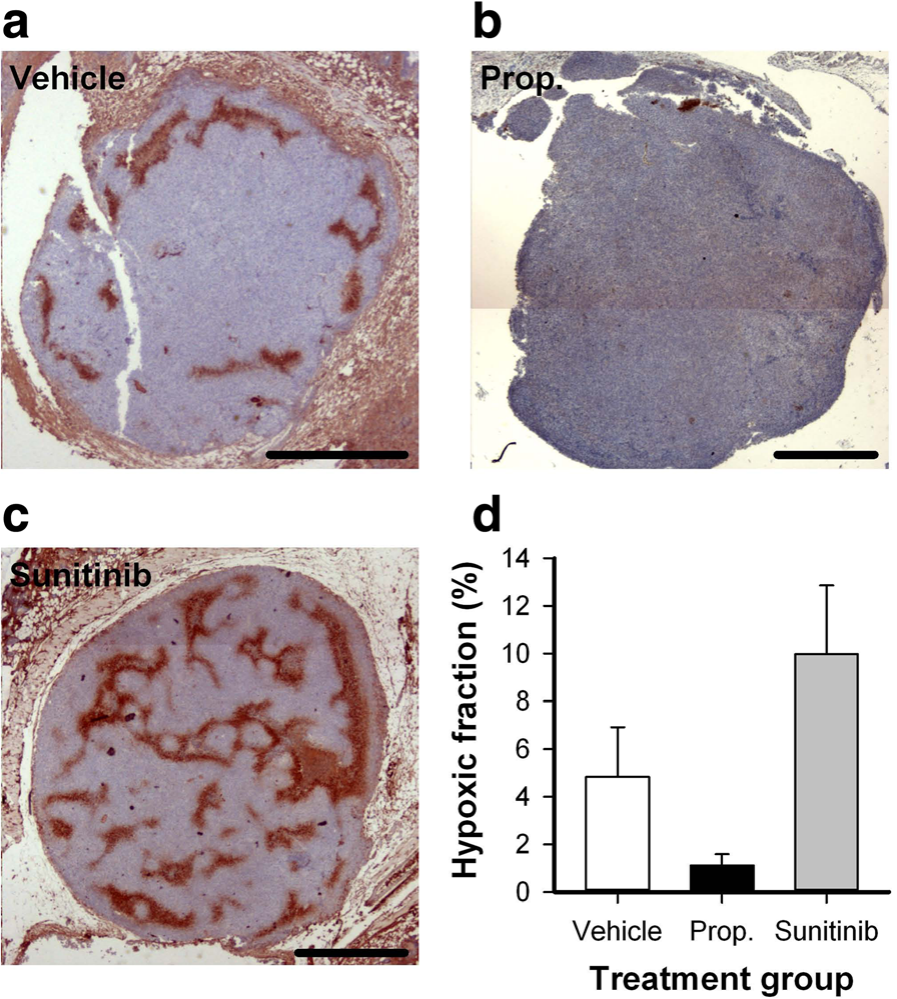
Tumor hypoxia. **a-c,** Immunohistochemical preparations stained for pimonidazole to visualize hypoxia of untreated (**a**), properdistatin-treated (**b**), and sunitinib-treated R-18 tumors (**c**). Scale bars, 1 mm. **d,** Hypoxic area fraction in untreated, properdistatin-treated, and sunitinib-treated R-18 tumors. Columns, means of 5–9 tumors; bars, SE

Figure 4a-c shows immunohistological preparations stained for hypoxia of representative untreated, properdistatin-treated, and sunitinib-treated tumors. Compared with untreated tumors, properdistatin-treated tumors generally showed less hypoxic regions, and sunitinib-treated tumors showed more extensive hypoxia. As a consequence, sunitinib-treated tumors showed significantly higher hypoxic fraction than properdistatin-treated tumors (Fig. 4; *P* = 0.028), demonstrating that these treatments affected tumor oxygenation fundamentally different.

## Discussion

Intravital microscopy allows assessment of the morphology and function of tumor vasculature [22], and was used to evaluate treatment-induced vascular effects in the current study. Morphological images were recorded with a spatial resolution that allowed detection of small-diameter tumor vessels (< 5 μm), and first-pass imaging movies were recorded with a temporal resolution (44.8 ms) that allowed BST measurement in individual vessels [20]. Intravital microscopy allows substantially higher spatial and temporal resolution than techniques used to image vasculature in human tumors, including angiography, but requires window chamber preparations and is thus limited to experimental studies [23].

Both properdistatin and sunitinib treatment inhibited angiogenesis in R-18 melanoma xenografts. Properdistatin selectively removed small-diameter vessels whereas sunitinib reduced the density of small- and large-diameter vessels similarly. Small-diameter vessels are expected to have a high geometric resistance to blood flow because the geometric resistance in tubes with laminar flow is inversely proportional to the vessel diameter to the fourth power [24]. Properdistatin treatment probably reduced the geometric resistance in the vascular networks by reducing the fraction of small-diameter vessels, whereas sunitinib treatment did not alter the fraction of small-diameter vessels and probably did not reduce the geometric resistance. In accordance with the effects on vascular morphology, properdistatin reduced BST, implying that the treatment improved vascular function, whereas sunitinib treatment did not change BST.

The treatment-induced effects on vascular morphology and function affected tumor oxygenation. Thus sunitinib-treated tumors showed higher hypoxic fractions than properdistatin-treated tumors. The sunitinib-induced increase in hypoxic fraction was probably a result of reduced oxygen supply caused by reduced vessel density. This finding is similar to our previous observations in sunitinib-treated A-07 melanoma xenografts [25]. Properdistatin-treated R-18 tumors also showed reduced vessel densities. However, in these tumors, improved vascular function probably compensated for the loss of tumor vessels and resulted in a reduction in hypoxic fraction compared with sunitinib-treated R-18 tumors.

Antiangiogenic agents targeting the VEGF-A pathway have been demonstrated to improve blood supply and oxygenation is some tumor models, and to induce hypoxia in others [3, 4, 10–12]. In the current study, we demonstrate that antiangiogenic agents targeting different angiogenic pathways affect vascular function and tumor hypoxia distinctly different in the same melanoma model. This has not been demonstrated previously and suggests that a specific clinical melanoma may experience improved blood supply and oxygenation if treated with one antiangiogenic agent, and increased hypoxic fraction if treated with another agent targeting a different angiogenic pathway.

The effect of antiangiogenic treatment in combination with immunotherapy or conventional chemotherapy is currently evaluated for patients with malignant melanoma [7]. Tumor hypoxia has been shown to induce immunosuppression and to reduce the effect of some chemotherapeutic drugs, suggesting that neoadjuvant antiangiogenic treatment may reduce the effect of immunotherapy and chemotherapy if the antiangiogenic agent induces hypoxia [8, 13, 26]. However, antiangiogenic treatment has been demonstrated to increase the effect of immunotherapy and chemotherapy in tumor models where antiangiogenic treatment improves vascular function and oxygenation [10, 27]. The current study suggests that neoadjuvant antiangiogenic treatment can reduce the extent of tumor hypoxia and thus increase the effect of immunotherapy and conventional chemotherapy if an appropriate antiangiogenic agent is used. However the study also suggests that neoadjuvant antiangiogenic treatment can induce hypoxia and reduce the effect of immunotherapy and chemotherapy if an inappropriate antiangiogenic agent is used. Consequently, the effect of antiangiogenic treatment should be monitored closely if antiangiogenic treatment is considered as neoadjuvant therapy in combination with immunotherapy or conventional chemotherapy. The current study demonstrates that assessment of vessel density is inadequate in determining treatment-induced effects on tumor oxygenation, because both properdistatin and sunitinib treatment caused a similar reduction in the overall vessel density but affected the extent of tumor hypoxia differently. This observation suggests that parameters describing vascular function are more appropriate for evaluating the effect of antiangiogenic treatment than vessel density. We have previously demonstrated that dynamic contrast-enhanced and diffusion-weighted magnetic resonance imaging is sensitive to sunitinib-induced tumor hypoxia and necrosis in melanoma xenografts, suggesting that these non-invasive imaging techniques may be used to monitor the effect of antiangiogenic treatment [25].

## Conclusions

Properdistatin and sunitinib treatment affected vascular morphology, vascular function, and tumor oxygenation fundamentally different. Sunitinib treatment reduced the density of both small- and large-diameter vessels and did not modify vascular function, whereas properdistatin treatment selectively removed small-diameter vessels and improved vascular function. As a consequence, sunitinib-treated tumors showed higher hypoxic fractions than properdistatin-treated tumors.

## Additional file

Additional file 1: First-pass imaging movie. First-pass imaging movie of a representative untreated R-18 tumor, illustrating how a bolus of 155 kD tetramethylrhodamine isothiocyanate-labeled dextran moves through the vascular network of the tumor. The movie lasts for 6.0 s and was recorded with a temporal resolution of 44.8 ms and a pixel size of 7.5 × 7.5 μm^2^. Tumor area is delineated by a solid black line. Scale bar, 1 mm. The BST image and the corresponding BST frequency distribution of the tumor are shown in Fig. 3a. (AVI 20979 kb)

## Abbreviations

BST: Blood supply time
GFP: Green fluorescent protein
VEGF: Vascular endothelial growth factor
